# Does the ultrasonic activation of calcium silicate-based sealers affect their physicochemical properties?

**DOI:** 10.1590/0103-6440202205100

**Published:** 2022-12-05

**Authors:** Isadora Ames Silva, Gabriel Barcelos Só, Theodoro Weissheimer, Aline Mendes, Lina Naomi Hashizume, Marcus Vinícius Reis Só, Ricardo Abreu Da Rosa

**Affiliations:** 1 Department of Endodontics, School of Dentistry, Rio Grande do Sul Federal University (UFRGS), Porto Alegre, RS, Brazil.; 2 Department of Preventive and Social Dentistry, Federal University of Rio Grande do Sul (UFRGS), Porto Alegre, RS, Brazil.

**Keywords:** bioceramic sealer, hydraulic calcium silicate-based sealers, ultrasonic activation, physicochemical properties

## Abstract

This study aimed to evaluate the influence of ultrasonic activation (UA) on the physicochemical properties of hydraulic calcium silicate-based sealers. Nine experimental conditions were created based on the hydraulic calcium silicate-based sealers (Bio-C Sealer, Sealer Plus BC and Bio Root RCS) and the ultrasonic activation (no activation [NA], 10 seconds, and 20 seconds). Then the experimental groups were BC-NA, BC-10, BC-20, SPBC-NA, SPBC-10, SPBC-20, BR-NA, BR-10, and BR-20. Activation was performed with an ultrasonic insert 20/.01. The mold for the physicochemical analysis was filled and evaluated according to the ANSI/ADA specification nº. 57: initial and final setting time, flow, radiopacity and solubility. Tests were also performed to evaluate pH and calcium ion release with experimental periods of 1, 24, 72, and 168 hours with a pH meter and colorimetric spectrophotometer. Data were analyzed by one-way analysis of variance and post-hoc Tukey tests. The significance level was set at 5%. The time of UA progressively delayed the initial setting time for all hydraulic calcium silicate-based sealers (p < 0.05). Twenty seconds of UA increased the mean flow values of Sealer Plus BC and Bio-C Sealer compared to NA (p < 0.05). UA did not influence the radiopacity and solubility of the tested sealers (p > 0.05). UA for 20 seconds enhanced the pH levels and the calcium ion release of Sealer Plus BC and Bio-C Sealer at 168h (p < 0.05). UA for twenty seconds interferes with some physicochemical properties of hydraulic calcium silicate-based sealers.

## Resumo

Este estudo teve como objetivo avaliar a influência da ativação ultrassônica (AU) nas propriedades físico-químicas de cimentos de silicato de cálcio. Nove condições experimentais foram criadas com base nos cimentos de silicato de cálcio (Bio-C Sealer, Sealer Plus BC e Bio Root RCS) e na ativação ultrassônica (sem ativação [SA], 10 segundos e 20 segundos). Os grupos experimentais foram BC-SA, BC-10, BC-20, SPBC-SA, SPBC-10, SPBC-20, BR-SA, BR-10 e BR-20. A ativação foi realizada com um inserto ultrassônico 20/.01. O molde para a análise físico-química foi preenchido e avaliado de acordo com a especificação ANSI/ADA nº. 57: tempo de presa inicial e final, escoamento, radiopacidade e solubilidade. Também foram realizados testes para avaliação de pH e liberação de íons cálcio com períodos experimentais de 1, 24, 72 e 168 horas com pHmetro e espectrofotômetro colorimétrico. Os dados foram analisados por análise de variância one-way e testes post-hoc de Tukey. O nível de significância foi estabelecido em 5%. O tempo de AU aumentou progressivamente o tempo de presa inicial para todos os cimentos de silicato de cálcio (p < 0,05). Vinte segundos de AU aumentaram os valores médios de escoamento de Sealer Plus BC e Bio-C Sealer em comparação com SA (P < 0,05). A AU não influenciou a radiopacidade e a solubilidade dos cimentos testados (P > 0,05). AU por 20 segundos aumentou os níveis de pH e a liberação de íons cálcio de Sealer Plus BC e Bio-C Sealer em 168h (P < 0,05). AU por vinte segundos interfere em algumas propriedades físico-químicas dos cimentos de silicato de cálcio.

## Introduction

The principles of endodontic therapy have been consolidated in the literature for almost a hundred years and are related to the proper sealing of the root canal system (RCS) after chemical-mechanical preparation ^1^. Endodontic sealers must penetrate the anatomical complexities of the RCS, such as irregularities, ramifications, isthmus, and dentinal tubules, promoting a better adaptation to the root canal walls through their physical and chemical interactions ^2^. The three-dimensional filling is essential for endodontic success and aims to provide the sealing of the canal system, entomb the remaining microorganisms, and favor the repair of the periapical tissues ^1^.

Hydraulic calcium silicate-based sealers, also known as bioceramic sealers, consist of a recent class of materials used in the medical and dental area and stand out for their biological similarity to hydroxyapatite, which confers excellent biocompatibility ^3^. In addition to being biocompatible and bioactive ^3^, bioceramic endodontic sealers have hydrophilic characteristics, making intracanal moisture necessary for their final setting, present antibacterial activity, and are radiopaque ^4^. The main drawback of these sealers is their high solubility ^3^.

Bio-C Sealer (Angelus Produtos Odontológicos, Londrina PR, Brazil), BioRoot RCS (Septodont, Saint-Maur-des-Fosses, France), and Sealer Plus BC (MK Life, Porto Alegre, RS, Brazil) are ready-to-use sealers. BioRoot RCS is a hydraulic calcium silicate-based sealer with a powder and liquid presentation. It has antimicrobial activity, bioactive capacity by releasing calcium ions ^5^, and adequate properties such as radiopacity, setting time, and pH, but high porosity and solubility ^5^. Ready-to-use bioceramic sealers have similar physicochemical characteristics, such as flow, setting time, radiopacity, and pH, but higher solubility according to the required standards ^6^ ^,^ ^7^. BioRoot RCS shows a shorter setting time and flowability than Bio-C Sealer ^8^. Both ready-to-use and powder/liquid bioceramic sealers are considered biocompatible and have bioactive potential ^3^.

Ultrasonic activation (UA) of the sealer aims to fill the irregularities, promote the sealer's penetration in difficult access, and improve the adaptation between the sealer and the root canal walls ^9^. Little is known about the influence of UA on the physicochemical properties of endodontic sealers. So far, only one study showed that UA influences the setting time and flow of a hydraulic calcium silicate-based sealer ^10^. Finally, the time required to activate the sealers is still varied in studies ^9^ ^,^ ^10^. For these reasons, this study aimed to evaluate the influence of the ultrasonic activation on the physicochemical properties of bioceramic sealers with different formulations (ready-to-use and powder/liquid) during ten and twenty seconds of activation. The null hypothesis of this study was that ultrasonic activation will not influence the physicochemical properties of hydraulic calcium silicate-based sealers.

## Material and methods

Three hydraulic calcium silicate-based sealers (Bio-C Sealer, Sealer Plus BC, and Bio Root RCS) were tested in the present study. The composition of each tested sealer is displayed in Table 1.

**Table 1:** Chemical compositions of the tested sealers.

Bio-C Sealer	Sealer Plus BC	BioRoot RCS	
		Powder	Liquid
Calcium Silicates	Calcium silicate	Tricalcium silicate	Water
Calcium aluminate	Zirconium oxide	Zirconium oxide	Calcium chloride
Calcium oxide	Tri-calcium silicate	Povidone	Polycarboxylate
Zirconium oxide	Propylene Glycol	-	-
Iron oxide	Calcium hydroxide	-	-
Silicon dioxide	-	-	-
Polyethylene Glycol	-	-	-

Sealer Plus BC and Bio-C Sealer are presented in a ready-to-use formulation. BioRoot RCS is presented in a powder/liquid formulation, and it was manipulated according to the manufacturer's instructions. All sealers were placed on an analytical balance and the weight was established in 0.3g. Therefore, nine experimental conditions were created based on the hydraulic calcium silicate-based sealers (Bio-C Sealer, Sealer Plus BC, and Bio Root RCS) and the ultrasonic activation (no activation, 10 seconds, and 20 seconds) as follow: BC-NA, BC-10, BC-20, SPBC-NA, SPBC-10, SPBC-20, BR-NA, BR-10, and BR-20. A 3 mL syringe was transversally sectioned with a heated instrument at 1 mL mark and filled with 0.3g of the sealer. A 20/.01 ultrasonic tip (E1 - Irrisonic Tip; Helse Dental Technology, São Paulo, Brazil) coupled in an ultrasonic device (P5 Newtron; Acteon Mount Laurel, USA) was immersed 5mm into the center of the sealer mass. Circular movements were performed at a power of 20% with the ultrasonic tip during the activation.

### Setting Time

Tests were performed under controlled temperature and humidity, 37°C ± 1°C and 95% ± 5%, respectively. Setting time was determined according to the specifications of ISO 6876:2012 ^6^ and ASTM C266-03 (ASTM C266-03). Three specimens per group (BC-NA, BC-10, BC-20, SPBC-NA, SPBC-10, SPBC-20, BR-NA, BR-10, BR-20), measuring 10mm in diameter and 2mm in height, were produced. Ultrasonic agitations were performed for each group, and the sealers were inserted into the matrix. Slightly moistened gauzes were placed below and over samples to provide the necessary moisture. This methodology was based on a previous study ^10^. After thirty seconds, a 100g Gilmore needle with a 2mm active tip was placed vertically on the sample surface. This procedure was repeated every 60 seconds until the sealer surface was no longer marked, defined as the initial setting time. The evaluation of the final setting time started immediately after determining the initial setting time. A 456.5g Gilmore needle with a 1mm active tip was positioned vertically on the sealer surface ^11^. The procedure was repeated as described for initial setting time. The same interval used to determine the initial setting time was used to determine the final setting time.

### Flow

The flow test was performed according to ISO 6876:2012 ^6^ specifications. For this test, 0.5 ± 0.005 mL of sealer was handled according to each group (n=3) and placed on a glass plate measuring 40mm (height) x 40mm (width) x 5mm (thickness) using a 1mL syringe. Then, another glass plate was placed over the sealer, and a 100g load was applied. After 10 minutes, each sample's most extensive and most minor diameters were measured with a digital caliper (Digimess, São Paulo, SP, Brazil), and the mean flow value was obtained. If the difference between the two diameters was higher than 1mm, the test was performed again. The final value was expressed in mm^2^ and was obtained from the mean values of three tests.

### Radiopacity

Following ISO 6876:2012 ^6^, three specimens per group were obtained. After handling, the sealers were introduced into silicon molds of 10 mm in diameter and 1 mm in height. After setting, the samples were placed on a periapical radiographic sensor next to an aluminum scale. This scale had a thickness ranging from 0.5 to 5 mm. Next, digital radiographs were taken using a Timex 70E X-ray device (Saevo, Ribeirão Preto, SP, Brazil) with an exposure time of 0.1 s. Images were analyzed with ImageJ software (Research Services Branch, National Institutes of Mental Health, Bethesda, MD, USA). The aluminum scale's gray levels (pixel densities) and a standardized area of 1.5 mm^2^ in the center of the samples were calculated concerning their mean values and standard deviations. The radiopacity value was determined according to the radiographic density, which was also converted into millimeters of aluminum (mm Al).

### Solubility

Three specimens with 10mm in diameter and 1mm in height ^6^ were produced for each group to determine solubility. The samples were weighed on an analytical balance (Shimadzu, Tokyo, Japan) with an accuracy of 0.001 g and then placed in Falcon tubes (Mano de mano Import, Osasco, São Paulo, Brazil) with 50 mL of distilled water. The specimens were inserted into the tubes using nylon thread, which allowed the sample to be hung and immersed in distilled water without touching the walls of the Falcon tubes during the entire experimental period. After setting, the specimens were removed from the molds, and all remaining particles were removed using a microbrush. The tubes were closed and conditioned at a temperature of 37°C ± 1°C and 95% ± 5% air humidity. After 168 h, specimens were removed from the tubes, gently washed with distilled water, dried with absorbent paper, placed in a dehumidifier for 24 hours, and weighed again to obtain their final weights. Solubility was obtained by calculating the weight loss after immersion (in grams).

### pH and calcium ion release

Five specimens of each group were produced from polyethylene tubes (10mm in length and 1mm inner diameter) with one of the ends closed, reproducing a closed system ^12^. The tubes were filled with sealer using a 1mL syringe. After filling, each specimen was placed in a 15ml tube containing 10ml of deionized water. The samples were stored at a controlled temperature of 37°C ± 1°C. The specimens were removed from the flask before the pH assessment, and the solutions were manually agitated for 5s. pH evaluation was performed with a digital pH meter (Digimed DM-22, San Paulo, SP, Brazil). The control was based on the pH values of deionized water in which no samples were immersed. The calcium ion release was obtained with a colorimetric method using arsenazo III ^13^. Both pH and calcium ion release were assessed after 1, 24, 72, and 168 hours.

### Statistical Analysis

The Shapiro-Wilk test was performed to evaluate the distribution of the data. Statistical analysis was performed using a one-way analysis of variance (ANOVA) and the Tukey test at a significance level of 5%.

## Results

Table 2 shows the results of initial and final setting times (minutes), flow (mm), radiopacity (mm/Al), and solubility (g) of the endodontic sealers assessed with and without UA.

**Table 2:** Means and standard deviations of initial and final setting times (minutes), flow (mm), radiopacity (mm/Al), and solubility (g).

Sealers	UA	Initial Setting Time	Final Setting Time	Flow	Radiopacity	Solubility
Bio-C Sealer	NA	80+1.11^Ca^	188.61+8.11^Ba^	37.21+0.14^Bab^	5.33+0.25^Aa^	-0.0338+0.0019^Aa^
	10 sec	85.66+1.01^Bb^	190.66+ 8.02^Ba^	39.22+0.08^Ba^	5.17+0.43^Aa^	-0.0341+0.0014^Aa^
	20 sec	95.66+1.17^Ab^	195.82+13.01^Aa^	43.07+0.13^Aa^	5.04+0.52^Aa^	-0.0358+0.0051^Aa^
Sealer Plus BC	NA	62.33+1.10^Cb^	168.33+8.50^Cb^	35.86+0.11^Bb^	5.11+0.06^Aa^	-0.0386+0.0021^Aa^
	10 sec	91+1.12^Ba^	185+6.76^Ba^	35.03+0.09^Bb^	5.07+1.10^Aa^	-0.0384+0.0019^Aa^
	20 sec	148.66+1.19^Aa^	196.66+11.17^Aa^	39.07+.12^Ab^	4.99+0.7^Aa^	-0.0398+0.0018^Aa^
BioRoot	NA	94+1.18^Ca^	125.32+8.43^Bc^	38.37+0.16^Aa^	5.05+0.47^Aa^	-0.0433+0.0027^Aa^
	10 sec	97+1.01^Ba^	127.91+7.85^Bb^	39.01+0.11^Aa^	5.02+0.22^Aa^	-0.0415+0.0022^Aa^
	20 sec	109+1.05^Ab^	132.33+.44^Ab^	38.34+0.09^Ab^	5.01+0.66^Aa^	-0.0421+0.0075^Aa^

Different capital letters denote a significant difference according to the UA of each sealer. Different lowercase letters denote a significant difference between the sealers for each activation time (α = 5%). NA = no activation, UA = ultrasonic activation.

### Setting Time

UA progressively delayed the initial setting time for all the hydraulic calcium silicate-based sealers (p < 0.05). Twenty seconds of UA delayed the final setting time for all the sealers compared with groups without activation (p < 0.05). However, ten seconds of UA delayed the Sealer Plus BC (p < 0.05). SPBC-NA and SPBC-20 showed the lowest and the highest initial setting time, respectively (p < 0.05). BioRoot RCS had the lowest final setting time in each experimental condition (p < 0.05).

### Flow

Twenty seconds of UA increased the mean values of flow for Sealer Plus BC and Bio-C Sealer compared to the same sealers without activation (p < 0.05). Ten seconds of UA did not alter the flow rate of the sealers (p < 0.05). The flow rate of BioRoot RCS was higher than Sealer Plus BC without activation (p < 0.05). After ten seconds of UA, Sealer Plus BC showed the lowest flow rate (p < 0.05). Bio-C Sealer showed the highest flow after twenty seconds of activation (p < 0.05).

### Radiopacity

UA did not influence the radiopacity of the sealers (p < 0.05). Bio-C Sealer, BioRoot RCS, and Sealer Plus BC sealers had similar radiopacity (p < 0.05).

### Solubility

UA did not affect the solubility of the tested sealers (p < 0.05). The hydraulic calcium silicate-based sealers showed similar solubility (p < 0.05).

### pH and Calcium Ion Release

Tables 3 and 4 present the pH and calcium ions release after different times of ultrasonic activation of the sealers in 1h, 24h, 72, and 168h. The pH values increased from 1 to 24 hours for all groups (P < .05), except for SPBC-NA and SPBC-20 (p < 0.05). From 24 to 72h, pH increased for BR-20, for SPBC-NA, and SPBC-20 (p < 0.05). Finally, from 72 to 168h, pH increased for BC-10, BC-20, and SPBC-20 (p < 0.05). UA for 20 seconds improved the pH levels and the calcium ion release of Bio-C Sealer and Sealer Plus BC in 168h (p < 0.05). BioRoot RCS had the highest pH in all periods evaluated, except BR-NA and BR-20 in 168h (p < 0.05). BioRoot RCS showed higher calcium ions release than Bio-C Sealer and Sealer Plus BC in all evaluated periods, regardless of the UA (p < 0.05).

**Table 3:** Means and standard deviation of pH after different times of ultrasonic activation of the sealers along the evaluation period.

Sealers	UA	1 hour	24 hours	72 hours	168 hours
Bio-C Sealer	NA	9.11±0.56 ^Ba*^	10.79±0.16 ^Aa†^	11.05±0.09 ^Aa*^	11.15±0.04 ^Ab†^
	10 sec	9.60±0.15 ^Ca*^	10.94±0.05 ^Ba†^	10.95±0.09 ^Bab*^	11.16±0.10 ^Ab†^
	20 sec	9.45±0.88 ^Ca*^	10.64±0.41 ^Ba*^	10.82±0.18 ^Bb*^	11.36±0.06 ^Aa*^
Sealer Plus BC	NA	9.97±0.45 ^Ba†^	10.41±0.16 ^Bb*^	10.96±0.24 ^Aa*^	10.99±0.15 ^Ab*^
	10 sec	10.10±0.12 ^Ba†^	10.78±0.05 ^Aa*^	10.92±0.02 ^Aa*^	10.78±0.34 ^Ab*^
	20 sec	10.36±0.21 ^Ca†^	10.55±0.15 ^Cb*^	10.85±0.02 ^Ba*^	11.69±0.15 ^Aa†^
BioRoot	NA	9.31±0.21 ^Bb*^	11.35±0.04 ^Aab§^	11.53±0.21 ^Aa†^	11.49±0.01 ^Aa§^
	10 sec	10.36±0.15 ^Ba†^	11.55±0.14 ^Aa§^	11.63±0.11 ^Aa†^	11.61±0.11 ^Aa§^
	20 sec	10.14±0.37 ^Ca†^	11.18±0.06 ^Bb†^	11.56±0.01 ^Aa†^	11.57±0.26 ^Aa*^

Capital letters compare the pH values in the line. Lowercase letters compare the effect of UA for each endodontic sealer. Different symbols denote differences between the sealers in each experimental condition in each evaluation period. (α = 5%). NA = no activation; UA = ultrasonic activation.

**Table 4:** Means and standard deviations of calcium ion release after different times of ultrasonic activation of the sealers along the evaluation period.

Sealers	UA	1 hour	24 hours	72 hours	168 hours
Bio-C Sealer	NA	53.67±5.70^Ba*^	418.51±205.1^Aa*^	580.76±69.26^Aa†^	550.62±52.85^Ab†^
	10 sec	59.86±6.10^Ca*^	459.96±96.27^Aba*^	399.29±69.11^Bb*^	584.45±124.0^Ab†^
	20 sec	61.23±6.55^Da*^	424.69±82.24^Ba*^	282.83±99.17^Cb*^	725.51±56.59^Aa*^
BioRoot	NA	453.22±66.65^Bb†^	1002.99±14.44^Ab†^	1027.53±38.42^Aab§^	1025.35±41.72^Aa§^
	10 sec	725.52±57.13^Ba§^	1034.63±23.74^Aa†^	1050.71±10.03^Aa†^	1040.83±47.35^Aa§^
	20 sec	758.92±93.00^Ba§^	1039.80±10.51^Aa†^	1011.86±8.66^Ab§^	1052.34±85.06^Aa§^
Sealer Plus BC	NA	387.92±96.99^Aa†^	257.46±84.60^Bb*^	413.37±76.57 ^Aa*^	434.76±37.51^Ab*^
	10 sec	278.53±68.70^Ba†^	427.96±51.23^Aa*^	437.62±15.43^Aa*^	408.65±76.87^Ab*^
	20 sec	342.05±59.60^Ca†^	361.47±30.57^Ca*^	425.77±12.05^Ba†^	918.61±23.28^Aa†^

Capital letters compare the calcium ion release in the line. Lowercase letters compare the effect of UA for each endodontic sealer. Different symbols denote differences between the sealers in each experimental condition in each evaluation period (α = 5%). NA = no activation; UA = ultrasonic activation.

## Discussion

New techniques and technologies are constantly being used to increase the success rate of endodontic treatment. UA increases the dispersion of the irrigant into the root canal system, improving cleaning, disinfection, and bacterial reduction ^14^. For these reasons, UA of endodontic sealers has been investigated in the last years ^9^, aiming to push the sealer toward the canal irregularities and dentinal tubules. However, it is still unclear whether the ultrasonic activation cause changes in the physicochemical properties of the sealers since there is little information on this topic, especially on hydraulic calcium silicate-based sealers. So, this study aimed to evaluate if UA would influence the physicochemical properties of hydraulic calcium silicate-based sealers. Based on the findings of this study, the null hypothesis was rejected. The ultrasonic activation influenced the physicochemical properties of hydraulic calcium silicate-based sealers.

Setting time is considered the time required for endodontic sealers to achieve their definitive properties. It is related to the sealer compounds, particle size, temperature, and relative humidity ^15^. It must be long enough to allow the insertion of the sealer into the root canals and the performance of the obturation technique ^16^. However, a long setting time can be a disadvantage, as most endodontic sealers have some degree of toxicity until their final setting ^17^. In addition, it can lead to apical leakage after endodontic treatment, leading to contamination by microorganisms through a sealer that has not yet been set ^18^.

Hydraulic calcium silicate-based sealers such as Bio-C Sealer and Sealer Plus BC have water-free thickeners, allowing them to be marketed as a ready-to-use, pre-mixed paste, making the final set of these sealers depend on the presence of moisture ^19^. Its components react with the water coming from the dentinal tubules; thus, the manufacturers' recommendation is not to excessively dry the canals with paper cones. However, the humidity inside the dentinal tubules varies ^20^, and it is not possible to be clinically measured, directly influencing the setting time of hydraulic calcium silicate-based sealers sold in ready-to-use packaging ^19^.

In this study, slightly moistened gauzes were placed over the samples to provide Sealer Plus BC and Bio-C Sealer with the necessary moisture to set. This model was based on a previous study ^10^ which used a paper filter and had similar setting times. The time of UA progressively delayed the initial setting time for all the hydraulic calcium silicate-based sealers. Also, twenty seconds of UA delayed the final setting time for all the sealers tested compared with no activation. High-frequency UA promotes turbulent flow and cavitation ^21^ and causes an increase in the temperature and pressure of the system, which probably generates radicals in the organic portion of the sealers, delaying the polymerization reaction and the setting time ^21^. BioRoot RCS showed the lowest final setting time in all experimental conditions, probably because of the aqueous vehicle in its composition, which accelerates the chemical reaction of the sealer's setting.

Regarding the results presented by the flow test, ten seconds of UA did not impact the flow rate for all the sealers tested. However, twenty seconds of UA increased the mean values of flow for the Sealer Plus BC and Bio-C Sealer compared to the same sealers without activation. UA did not influence the flow of the BioRoot RCS sealer. This result can probably be explained due to the pronounced heat generated when a more prolonged ultrasonic activation was performed, which can increase the flow of sealers in paste-paste or ready-to-use presentations ^22^. In addition, different vehicles compose the hydraulic calcium silicate sealers tested in this study. Propylene glycol and polyethylene glycol are viscous vehicles presented in the Sealer Plus BC and Bio-C Sealer.

In contrast, BioRoot RCS has an aqueous vehicle in its composition, which confer greater flow even without UA when compared to the ready-to-use sealers. In addition, Bio-C Sealer showed the highest flow after twenty seconds of UA, probably because it presents the smallest particle size among the tested sealers. Nevertheless, all sealers reached the standards determined by ISO 6876:2012, which determines a flow rate of at least 20mm ^6^.

Endodontic sealers must have adequate radiopacity to be distinguishable from adjacent anatomical structures and to allow visualization of the quality of root canal filling. UA did not influence the radiopacity of the sealers. According to the ISO standards ^6^, an endodontic sealer must have a radiopacity equivalent to 3 mm of the aluminum scale. All sealers were within the required standards.

Solubility is related to the weight loss of the material when immersed in water ^16^. Sealers must remain within the established standards to avoid voids capable of allowing the infiltration of microorganisms and reinfection ^23^. All tested sealers presented higher solubility than the minimum recommended by ISO standards ^6^, regardless of the UA. The high solubility of bioceramic sealers occurs because of hydrophilic nanometric particles, which increase the surface area and allow more liquid molecules to meet the sealer ^17^. Bio-C Sealer present on its composition 2 μm particles, Sealer Plus BC 3 to 6 μm particles, BioRoot RCS 2 to 10 μm. The high solubility of these sealers is still one of the significant disadvantages of hydraulic calcium silicate-based sealers.

Assessing the sealers' pH is essential because it is related to the alkalization promoted by each sealer over time ^3^. Alkaline pH enhances antibacterial activity, which may have remained viable after chemomechanical preparation and could induce or maintain periapical disease ^4^. Furthermore, it can increase the sealers' osteogenic potential, biocompatibility, and mineralized components deposition ^24^. The pH values and calcium ion release are closely related to the solubility of sealers. The high solubility of calcium silicate-based sealers is considered a disadvantage; however, its bioactive potential through the release of calcium ions is a consequence of this solubility even after the final setting ^3^.

This study showed that the bioceramic sealers had high values of pH and calcium ion release, which increased or remained stable over time. SPBC-20 and BC-20 showed an increase in pH levels and calcium ion release in 168h (p < 0.05). Both sealers present a viscous vehicle. For this reason, it could be hypothesized that the UA for 20 seconds of bioceramic sealers with viscous vehicles could be an exciting strategy during clinical practice, especially in cases of a slow alkalinization of the medium such as root resorptions or dental trauma is required. This approach could promote better alkalinization of the medium with some impact on the disinfection process after root canal filling. Controversially, BioRoot RCS was not influenced by the UA (p < 0.05) but still presented higher pH and calcium ion release values since the first experimental times when compared to other calcium silicate-based sealers (p < 0.05). These results are corroborated by a previous study ^25^, and it can probably be explained due to its pronounced ionic diffusion in the aqueous vehicle.

## Conclusion

Based on the findings of this study, it can be concluded that twenty seconds of UA interfere with some physicochemical properties of calcium silicate-based sealers, improving the flow, pH, and calcium ion release of ready-to-use sealers (Sealer Plus BC and Bio-C Sealer). The initial and final setting time of calcium silicate-based sealers was progressively delayed the longer the UA.
